# Does the Punishment Fit the Crime (and Immune System)? A Potential Role for the Immune System in Regulating Punishment Sensitivity

**DOI:** 10.3389/fpsyg.2020.01263

**Published:** 2020-06-22

**Authors:** Jeffrey Gassen, Summer Mengelkoch, Hannah K. Bradshaw, Sarah E. Hill

**Affiliations:** Department of Psychology, Texas Christian University, Fort Worth, TX, United States

**Keywords:** punishment sensitivity, inflammation, cytokines, reward sensitivity, risk sensitive foraging, criminal justice

## Abstract

Although the criminal justice system is designed around the idea that individuals are invariant in their responses to punishment, research indicates that individuals exhibit a tremendous amount of variability in their punishment sensitivity. This raises the question of why; what are the individual- and situation-level variables that impact a person’s sensitivity to punishment? In the current research, we synthesize theory and research on inflammation, learning, and evolutionary biology to examine the relationship between inflammatory activity and sensitivity to punishment. These theories combine to predict that inflammatory activity – which is metabolically costly and reflects a context in which the net payoff associated with future oriented behaviors is diminished – will decrease sensitivity to punishment, but not rewards. Consistent with this hypothesis, Study 1 found that in U.S. states with a higher infectious disease burden (a proxy for average levels of inflammatory activity) exhibit harsher sentencing in their criminal justice systems. Studies 2 and 3 experimentally manipulated variables known to impact bodily inflammatory activity and measured subsequent punishment and reward sensitivity using a probabilistic selection task. Results revealed that (a) increasing inflammation (i.e., completing the study in a dirty vs. clean room) diminished punishment sensitivity (Study 2), whereby (b) administering a non-steroidal anti-inflammatory drug, suppressing inflammatory activity, enhanced it. No such changes were found for reward sensitivity. Together, these results provide evidence of a link between the activities of the immune system and punishment sensitivity, which may have implications for criminal justice outcomes.

## Introduction

Learning about the consequences of one’s actions and using this knowledge to maximize rewards and avoid threats is critical to survival and reproduction (Bouton, 2007; Balleine, 2011; Jean-Richard-Dit-Bressel et al., 2018). This fundamental process, often referred to as instrumental (or operant) conditioning, involves increasing or decreasing the frequency of behaviors that have been previously reinforced (i.e., that are followed by reward) or punished (i.e., that are followed by an adverse outcome), respectively (Thorndike, 1898; Skinner, 1963; Staddon and Cerutti, 2003; Bouton, 2007).

Although fundamental to survival and reproduction, individuals vary in their responses to rewards and punishments (e.g., Farmer, 2005; Kim et al., 2015; Jean-Richard-Dit-Bressel et al., 2018). These differences can have a meaningful impact on one’s likelihood of experiencing a variety of negative health and behavior problems (Potts et al., 2006; Kim et al., 2015; Harms et al., 2018). For example, research finds that people low in punishment sensitivity are more prone to substance abuse issues and problematic gambling than those higher in punishment sensitivity (e.g., Jean-Richard-Dit-Bressel et al., 2018; Smith and Laiks, 2018; Sistad et al., 2019). Others find that punishment sensitivity is altered in a number of psychological disorders (e.g., schizophrenia, attention deficit hyperactivity disorder; Farmer, 2005; Jappe et al., 2011). Understanding the factors that contribute to differences in punishment sensitivity therefore delineates an important area for research into human psychological functioning in both clinical and non-clinical populations.

Here, we use insights from psychoneuroimmunology (Maier and Watkins, 1998; Banks, 2005; Dantzer and Kelley, 2007; Lasselin et al., 2017; Draper et al., 2018) and risk-sensitive foraging theory (RSFT; Real and Caraco, 1986; Lima and Dill, 1990; Houston, 1991; McNamara and Houston, 1992) to examine the role that signaling by the immune system plays in modulating one’s sensitivity to punishment. Combining these insights yields the hypothesis that bodily inflammatory activity will cause strategic resource allocation shifts that decrease sensitivity to punishment, but not rewards (Muehlenbein et al., 2010; Lacourt et al., 2018; Wang et al., 2019). Specifically, we predict that inflammation will predict reduced punishment sensitivity because an inflammatory event reflects an internal, physiological context in which (a) the expected net payoff of future-oriented behaviors lower than what it is in its absence (due to the relatively diminished probability of survival; see, e.g., Gassen et al., 2019a, b; Gassen and Hill, 2019), and (b) one’s ability to suppress dominant responses is compromised due to the immunometabolic constraints that occur in the context of inflammation (see e.g., Lacourt et al., 2018; Treadway et al., 2019).

We tested our predictions in a series of three studies using a variety of methods and measures. First, we examined the relationship between environmental conditions that promote inflammatory activity (i.e., high vs. low environmental pathogen prevalence) and the tendency to use harsher punishments for criminal offenders. Because punishment severity typically varies as an inverse function of sensitivity (with greater insensitivity requiring harsher punishments to decreased the frequency of a behavior; see, e.g., Jean-Richard-Dit-Bressel et al., 2018; Marchant et al., 2018), we predicted that pathogen prevalence would predict the use of harsher punishments. Next, we conducted two experiments in which we experimentally manipulated factors known to increase (Study 2) or suppress (Study 3) inflammatory activity and measured subsequent sensitivity to punishment and rewards. We predicted that contexts that elicit an increase in inflammation would diminish punishment sensitivity, and that those that reduce inflammatory activity (i.e., aspirin) would enhance it. We predicted that no such changes would be found for sensitivity to rewards.

The ability to modify one’s behavior in response to rewards and punishment is critical to survival and is found in organisms as simple as mollusks (e.g., Nargeot and Simmers, 2011) and as complex as humans (for review see Shanks, 1993). Instrumental (or operant) conditioning refers to the process by which individuals learn contingencies between their behavior and positive or negative experiences and then use this information to maximize rewards and avoid punishment in the future (Thorndike, 1898; Skinner, 1963; Staddon and Cerutti, 2003; Bouton, 2007; Balleine, 2011; Jean-Richard-Dit-Bressel et al., 2018).

Despite being critical for survival and reproduction, individuals differ considerably in their sensitivity to rewards and punishment (Farmer, 2005; Kim et al., 2015; Jean-Richard-Dit-Bressel et al., 2018). For example, several studies have identified certain personality traits that are related to individual differences in reward and punishment sensitivity. Increased reward sensitivity is related to higher trait sensation-seeking (Scott-Parker et al., 2013) and lower agreeableness and conscientiousness (Mitchell et al., 2007). Others find that punishment sensitivity is lower in individuals who report high (compared to low) levels of impulsivity (Potts et al., 2006).

More recently, researchers have begun to explore the neurobiological underpinnings of sensitivity to punishment and reward. This research has revealed that individual differences in reward and punishment sensitivity are reflected in neural responses to appetitive and aversive stimuli. For example, one study found that individuals high in trait reward sensitivity (compared to those lower in reward sensitivity) exhibited increased activity in the right ventral striatum in response to monetary rewards, while individuals high in trait punishment sensitivity (compared to those lower in punishment sensitivity) exhibited increased activity in the right lateral orbitofrontal cortex in response to monetary punishment (Kim et al., 2015). These patterns suggest that reward and punishment sensitivity are regulated by distinct brain regions. Others find that lower punishment sensitivity is associated with reduced activity in the anterior insular and anterior cingulate cortices (Dong et al., 2011; Santesso et al., 2011; Palminteri et al., 2012), which is consistent with the view that brain areas involved in emotional processing and self-regulation play important roles in modulating responses to aversive stimuli.

Recent research suggests that the activities of the immune system may also play a role in regulating reward and punishment sensitivity. Although it was long believed that the immune system’s contribution to behavior was limited to its impact on the biological events involved in fighting infection and promoting recovery (Janeway and Medzhitov, 2002; Matzinger, 2002; Iwasaki and Medzhitov, 2010), it is now well-appreciated that the immune system also influences the activities of the nervous system (for review see Benveniste, 1992; Banks, 2005; Erta et al., 2012). For example, in the context of an acute inflammatory response, cytokines – which are signaling molecules released by the immune system – induce a state of sickness behavior, characterized by social disinterest, fatigue, and reduced grooming. This behavioral constellation functions to conserve energetic resources for the metabolically costly immune response and promote recovery (Aubert, 1999; Dantzer and Kelley, 2007; Medzhitov et al., 2012; Lopes, 2014). In addition to playing an important role in shaping behaviors that occurs in the context of sickness, cytokines are also understood to be instrumental in the processes that regulate mood (Maier and Watkins, 1998), executive functioning (e.g., Dozmorov et al., 2018; Gassen et al., 2019a, b), sleep (Opp, 2005), sensation (Miller et al., 2009), and many other processes in healthy people (Kipnis, 2018; Gassen and Hill, 2019).

Given the important role that signaling by the immune system can play in modulating decision-making and behavior, researchers have begun to explore the possibility that the immunometabolic shifts that occur in the context of inflammatory activity may have an impact on choice behaviors that require effortful control (for a review see Lacourt et al., 2018; Treadway et al., 2019). Decreased investment in behaviors requiring effort is hypothesized to occur in the context of inflammation because proinflammatory cytokines induce immunometabolic shifts – including increased overall metabolic rate and a reprogramming of immune cells to rely on swifter, yet less efficient energy production pathways (e.g., glycolysis) – that constrain energetic resources (Muehlenbein et al., 2010; O’Neill et al., 2016; Lacourt et al., 2018; Treadway et al., 2019; Wang et al., 2019). As a result, individuals experiencing an inflammatory response are less able (and willing) to exert effort in pursuit of potential rewards. Further, given that inflammation reflects an internal bodily context in which the probability of survival is relatively diminished, the expected net return on investment in future-oriented pursuits, such as learning, is lower than what it is in the absence of an inflammatory event (Gassen et al., 2019a, b; Gassen and Hill, 2019). Given the importance of taking advantage of immediately available, or low-effort resource opportunities during a metabolically costly immune response, sensitivity to immediate or low-cost rewards is expected to remain intact in the context of inflammation (for a review see Treadway et al., 2019).

Consistent with these ideas, one recent study in mice found that injection with lipopolysaccharide (LPS) – an endotoxin that elicits an increase in inflammation – reduced animals’ willingness to work for grain (low value reward), but not chocolate pellets (high reward value) (compared to control group injected with saline; Vichaya et al., 2014). In other words, although the mice’s tendency to exert effort for low-value rewards decreased in the context of inflammation, reward sensitivity, *per se*, appeared to be unchanged. Research using humans as participants also supports the hypothesis that increases in inflammation diminish one’s willingness to expend effort to obtain rewards, but have less of an impact on reward sensitivity (e.g., Lasselin et al., 2017; Draper et al., 2018; Boyle et al., 2019; for exception see Eisenberger et al., 2010). For instance, one study found that participants administered LPS (compared to the control group administered saline) were less willing to expend effort to obtain high-effort rewards (i.e., rewards that required a greater percent maximal voluntary contraction in a handheld dynamometer to obtain). However, reward sensitivity – or the preference for higher value rewards – was unaffected by LPS administration (Draper et al., 2018). Together, this research suggests that increases in inflammation tend to reduce individuals’ willingness to expend effort in pursuit of rewards, while reward sensitivity, *per se*, is preserved in this context (for an exception, see Harrison et al., 2016).

Does inflammatory activity lead to comparable effort-minimizing shifts in punishment sensitivity? The existing results are mixed. For example, research in rodents finds that experimental inflammatory challenges reliably impair both passive and active avoidance behavior (e.g., to avoid a foot shock), as well as contextual fear conditioning (Pugh et al., 1998; Patil et al., 2003; Sparkman et al., 2005; Kohman et al., 2007). Additional studies have demonstrated that infected animals tend to exhibit dampened antipredator responses compared to non-infected animals (e.g., mice: Kavaliers and Colwell, 1995; birds: Adelman et al., 2017; frogs: Preston et al., 2014; Rae and Murray, 2019). Together these findings support the prediction that sensitivity to aversive stimuli unrelated to the immediate threat of illness should decrease in the context of inflammation, when energetic resources are constrained and the shadow of the future shortens.

Nonetheless, direct experimental research on humans suggests that punishment sensitivity may be heightened in the context of inflammation. For example, in one study, researchers examined the impact of typhoid vaccine-induced inflammation on performance on a probabilistic selection task (PST; Harrison et al., 2016). The results of this study revealed that heightened inflammation was associated with enhanced punishment sensitivity 2.5–3.5 h post-manipulation. These results are consistent with research finding that large increases in inflammation induce hyperalgesia and depressed mood 2–3 h after the eliciting event (e.g., Harrison et al., 2009; Wegner et al., 2014). Others find no impact of states known to be linked with elevated inflammatory activity, such as depression (e.g., Kunisato et al., 2012) and acute stress (e.g., Berghorst et al., 2013), on punishment sensitivity.

In the current set of studies, we sought to add to this body of research by examining the relationship between immune activation and punishment-related decision-making (a) at a population level by examining the role that infectious disease burden plays in predicting crime rates and criminal justice outcomes across the United States (Study 1), and (b) at the individual-level, using experimental manipulations designed to increase (Study 2) and suppress (Study 3) inflammatory activity on large, highly powered samples. To test the relationship between inflammatory activity and punishment sensitivity in the two laboratory studies (Studies 2–3), we used the well-validated probabilistic selection task (PST). The PST has been used for almost two decades to study how individuals learn from positive and negative feedback (Frank et al., 2004). Studies utilizing the PST to investigate instrumental learning have revealed that deficits in learning from positive or negative experiences underlie a number of different neurological disorders, such as depression (Kunisato et al., 2012), schizophrenia (Waltz et al., 2007), and Parkinson’s disease (Frank et al., 2004). For example, research using this task finds that reward-sensitive learning is impaired in depressed and schizophrenic participants, while punishment-based learning is unaffected by these conditions (Waltz et al., 2007; Kunisato et al., 2012). Others find that Parkinson’s patients off medication are better at learning from punishments than rewards, but this bias is reversed for patients taking medication targeting dopaminergic signaling, likely due to the effects that these drugs have on dopamine-basal ganglia interactions (Frank et al., 2004). We predicted that environmental factors that increase inflammation would predict decreases in sensitivity to punishment (Study 2), while pharmacological manipulations designed to decrease inflammation would predict increases in sensitivity to punishment (Study 3), as measured via the PST, while in both cases, reward sensitivity would remain unaffected (Lasselin et al., 2017; Draper et al., 2018; Boyle et al., 2019; for exception see Harrison et al., 2016). All data analyzed in the current research can be found on the Open Science Framework (DOI: 10.17605/OSF.IO/29H7V).

## Study 1: State-Level Pathogen Prevalence and Punitive Justice

In Study 1, we sought to examine the hypothesized relationship between inflammatory activity and punishment sensitivity using data available from the U. S. Justice System. We predicted that environmental factors that increase immune activation – such as higher infectious disease burden (e.g., Zhu et al., 1999; Gattone et al., 2001; Nazmi et al., 2010; Thompson et al., 2014; Ferrucci and Fabbri, 2018) – would be associated with the use of harsher punishments for misconduct. Specifically, we predicted that U.S. states with a higher infectious disease burden would engage in harsher, more punitive sentencing than states with lower infectious disease burden to counter the reduced punishment sensitivity that is predicted to occur in the context of immune activation. This prediction is consistent with research finding that harsher punishments are required to modify the behavior of individuals with lower punishment sensitivity (compared to those with higher punishment sensitivity; Jean-Richard-Dit-Bressel et al., 2018; Marchant et al., 2018).

### Materials and Methods

To test our prediction that higher infectious disease burden would yield harsher, more punitive sentencing, we accessed public data bearing on each U.S. state’s (a) infectious disease burden, (b) crime rate, and (c) incarceration rates and relative sentencing harshness. We describe these measures below, and additional information about each variable is displayed in Table 1.

**TABLE 1:** Summary of variables and source of data for Study 1.

Measure	Source	Description
Infectious disease burden	Fincher and Thornhill, 2012	Population-adjusted infectious illnesses for each state from 1993 to 2007 (z-score)
Crime rate	FBI uniform crime reporting data	Population-adjusted crime rates (by category) for 2016 in each state
Incarceration and community corrections	Bureau of Justice Statistics (NCJ 251211)	Population-adjusted rates of individuals incarcerated or undergoing community supervision for 2016 in each state
Punishment rate	The Pew Charitable Trusts	Proprietary metric for assessing crime type-adjusted punishment severity for 2013 in each state
Income	U.S. Census Bureau (2018) American Community Survey	Median household income in each state (2013–2017)
Education level		Percent of state who did not complete high school (2013–2017)
Age		Percent of state aged 18–39 (2013–2017)
Income inequality		Gini coefficient for each state (2013–2017)
Sex		Percent of state that was male (2013–2017)

**FBI, Federal Bureau of Investigation; NCJ, National Criminal Justice.**

#### Infectious Disease Burden

To measure each state’s infectious disease burden, we utilized previously published data (Fincher and Thornhill, 2012) obtained from the Center for Disease Control’s (CDC) annual *Morbidity and Mortality Weekly Report’s* “Summary of Notifiable Diseases, United States” (1993–2007)^1^. To compute a total parasite stress score for each year, the researchers adjusted the number of infectious disease cases to account for each state’s population size, and computed a standardized *z*-score of each state’s infectious disease rate across the 15-year span. A list of all diseases included in the index can be found in the electronic supplemental material for the original article (Fincher and Thornhill, 2012; *ES 3*). This index has been used in several studies as a measure of state-level infectious disease rate (e.g., Eppig et al., 2011; Thornhill and Fincher, 2011; Harrington and Gelfand, 2014).

#### Crime Rate

To determine each state’s crime rate, we downloaded arrest records in each state from the Federal Bureau of Investigation’s (FBI) Uniform Crime Reporting (UCR) public data^2^. We used the data from the year 2016 as this was the most recent year available for the data representing our target dependent measure (see Incarceration Rates and Punitive Justice below). Arrest records were classified as follows: violent crime (murder and non-negligent manslaughter, rape, robbery, and aggravated assault; per FBI classification in data set), property crime (burglary, larceny-theft, motor vehicle theft, arson; per FBI classification in data set), and all other crime (other assaults, forgery and counterfeiting, fraud, embezzlement, stolen property, vandalism, weapons, prostitution and commercialized vice, sex offenses other than rape/prostitution, drug, gambling, offenses against family, driving under the influence, liquor law violations, drunkenness, disorderly conduct, vagrancy, all other non-traffic offenses, suspicion, and curfew and loitering). We computed population-adjusted crime rates by dividing the number of arrests in each category by the state’s total population. Note that in these data, multiple arrests from the same individual are counted as multiple data points. Thus, repeat offenders could introduce bias into this metric.

#### Incarceration Rates and Punitive Justice

We accessed data bearing on each state’s rates of persons supervised by adult correctional systems from the Bureau of Justice Statistics’ (BJS) “Correctional Populations in the United States, 2016” report (NCJ 251211; Kaeble and Cowhig, 2016). These data include a breakdown of both the state’s rate per 100,000 people in the total population currently undergoing community supervision programs (i.e., probation or parole), as well as rate per 100,000 people in the total population currently incarcerated.

As an additional measure of a state correctional system’s tendency toward punitive measures, we also accessed data from The Pew Charitable Trusts, who computed a “punishment rate” for each state. This nuanced metric adjusts the size of the prison population in each state accounting for a severity-weighted crime rate in that state [a scale that weighs each crime (drug offenses not included) by the average imprisonment term served for that offense]. Higher values represent more severe punishments for the same crime. Data used in the current analysis are from 2013, the latest available for this metric. More information about how the metric is computed can be found in a brief published by The Pew Charitable Trusts (2016).

#### Covariates

Because a number of other state-level variables likely also contribute to crime and incarceration rates, we also collected information on each state’s median household income, education level (i.e., percent who did not complete high school), percent of population aged 18–39, income inequality (i.e., Gini coefficient), and the percentage of the state’s population who were male. These data were averages from the years 2013 to 2017 and were taken from the American Community Survey conducted by the U.S. Census Bureau.

### Data Analysis Plan

With the exception of the violent crime rate, all variables approximated a normal distribution. Violent crime rate was slightly positively skewed, and as such we estimated a regression model using robust maximum likelihood (MLR) estimation in MPlus statistical software (Version 8, Muthén and Muthén, 2012), a method that is robust to non-normal data distributions and heteroscedasticity (Kline, 2016; Maydeu-Olivares, 2017). As an additional measure of parameter reliability, we generated 95% credibility intervals (CIs; interpreted in the same fashion as confidence intervals) for each effect using Bayesian estimation with non-informed priors in Mplus. Bayesian estimation provides a number of advantages over frequentist methods, namely that it does not assume normal parameter distribution (Muthén, 2010). We considered parameters significant only if they had a *p* < 0.05 and the CI for the effect did not contain 0.

First, as an exploratory analysis, we tested whether infectious disease burden predicted total crime, violent crime, and/or property crime rates using a multivariate regression analysis. Crime rates were converted to z-scores to fix model convergence issues. Although we did not have clear predictions about relationships between these different types of crime variables, we included this analysis as it may be of interest to readers. Next, as our first measure of states’ tendency toward harsh, punitive sentencing, we tested whether infectious disease burden predicted (a) rate of persons in community supervision and (b) rate of persons incarcerated, while controlling for the state’s rates of violent, property, and other crime. We predicted that greater infectious disease burden would predict higher rates of incarceration (i.e., harsher, more punitive sentencing) and lower rates of community supervision (i.e., less harsh sentencing). Finally, we tested in a separate model whether infectious disease burden predicted each state’s punishment rate (The Pew Charitable Trusts, 2016), a metric that already accounts for crime frequency and severity (see section “Materials and Methods” for more details). We tested each model a second time controlling for covariates (see section “Materials and Methods” and Table 1 for covariates). For analyses with one predictor, we report effect sizes as *R*^2^. For analyses with multiple predictors, we report Cohen’s *f*^2^, a measure of local effect size (Selya et al., 2012).

### Results and Discussion

#### Infectious Disease Burden and Crime Rates

**FIGURE 1:**
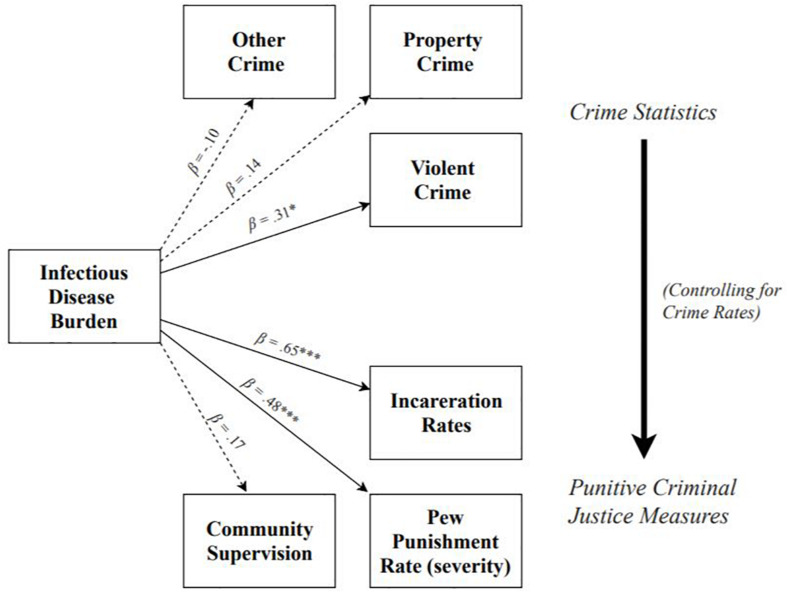
Summary of models testing effects of infectious disease burden on population-adjusted rates of crime, incarceration, community supervision, and Pew metric of punishment severity. Effect of infectious disease burden on criminal justice outcomes shown controlling for crime rates. Dotted lines denote non-significant paths; solid lines denote significant paths. *p < 0.05, **p < 0.01, ***p < 0.001.

See Figure 1 for a summary of Study 1 results. Results revealed that higher infectious disease burden predicted higher rates of violent crime, β = 0.31, *SE* = 0.16, *t* = 2.00, *p* = 0.045, 95% CI = [0.05, 0.49], *R*^2^ = 0.10, but not property crime, β = 0.14, *SE* = 0.16, *t* = 0.93, *p* = 0.35, 95% CI = [-0.19, 0.40], *R*^2^ = 0.02, or other crime, β = −0.10, *SE* = 0.12, *t* = −0.78, *p* = 0.44, 95% CI = [−0.35, 0.21], *R*^2^ = 0.01. When adjusting for covariates (see section “Materials and Methods” for full list), greater infectious disease burden significantly predicted higher rates of both violent crime (*p* = 0.035, *f*^2^ = 0.11), as well as property crime (*p* = 0.02, *f*^2^ = 0.08), but not other crime (*p* = 0.23, *f*^2^ = 0.03).

#### Infectious Disease Burden and Incarceration Rates

Results revealed that, adjusting for each state’s rate of violent, property, and other crime, greater infectious disease burden significantly predicted higher rates of incarceration, β = 0.65, *SE* = 0.08, *t* = 7.72, *p* < 0.001, 95% CI = [0.40, 0.75], *f*^2^ = 0.72, but not rates of community supervision, β = 0.17, *SE* = 0.14, *t* = 1.25, *p* = 0.21, 95% CI = [−0.27, 0.48], *f*^2^ = 0.03. Controlling for covariates did not change the pattern or significance of the effects of infectious disease burden on either incarceration rates (*p* < 0.001; *f*^2^ = 0.36), or community supervision rates (*p* = 0.47; *f*^2^ = 0.01), but did lower the effect sizes.

#### Infectious Disease Burden and Punishment Rate (The Pew Charitable Trusts)

Results revealed that, adjusting for each state’s rate of violent, property, and other crime, greater infectious disease burden significantly predicted a higher punishment rate, β = 0.48, *SE* = 0.14, *t* = 3.545, *p* < 0.001, 95% CI = [0.14, 0.63], *f*^2^ = 0.25. Controlling for covariates did not change the pattern or significance of this effect (*p* < 0.001; *f*^2^ = 0.18), but did slightly lower the effect size.

The results of Study 1 suggest that, controlling for crime rate, states with a greater infectious disease burden tend to employ harsher, more punitive criminal justice measures. However, infectious disease burden does not predict rates of community supervision. These results provide key initial support for the hypothesis that environmental factors that elicit heightened immune activity may promote the use of particularly harsh punishments. While we propose that reliance on harsh punishments may represent a response to the reduced punishment sensitivity that occurs in this context, we cannot directly test this prediction with the current data. We discuss this topic further in the general discussion.

## Study 2: Situational Antecedents to Inflammation and Sensitivity to Reward and Punishment

Study 1 found that the criminal justice systems of U.S. states with a higher infectious disease burden tended to utilize harsher punishments than states with a lower infectious disease burden. To investigate if these harsher punishments may be a reaction to decreased punishment sensitivity exhibited by those with heightened inflammation, in Study 2, we manipulated cues of pathogens in one’s immediate environment and measured participants’ (a) inflammation and (b) reward and punishment sensitivity. We predicted that sensitivity to punishment would vary as a function of inflammatory activity, with contexts eliciting heightened inflammation leading to a decrease in punishment sensitivity. Specifically, we predicted that participants completing the study in a dirty room (compared to those completing the study in a clean room) would have (a) higher levels of IL-1β, a key proinflammatory cytokine and (b) lower punishment sensitivity than those completing the study in a clean room. However, we predicted that there would be no differences between the two groups in reward sensitivity.

### Materials and Methods

#### Participants

A total of 138 participants (75 women, *M*_*age*_ = 19.00 years, *SD*_*age*_ = 1.15, age range: 17–24 years) completed the study in exchange for partial course credit. Participants were instructed not to eat or drink anything for at least 2 h prior to their session; smokers were told not to smoke the for at least 5 h prior to their session. Only participants who reported being free of chronic physical and psychological conditions were recruited for the study.

#### Design and Procedure

This study was approved as ethical by Texas Christian University’s Institutional Review Board (DRB 1709-11). Sessions were conducted in small groups (2–6 participants). All sessions took place in the afternoon (between 1:00 and 3:00 p.m.). Participants in each group first entered a clean laboratory and provided informed consent. Participants were told that they would be participating in a study about how bodily states influence perceptions of social and non-social stimuli. To support the ruse, participants were asked a variety of questions about their current bodily state (e.g., “How stressed do you feel at this moment?”). Participants next provided a baseline 5 mL saliva sample via passive drool into scintillation vials (Wheaton Industries, Millville, NJ, United States). Participants were then escorted to either (a) a separate computer lab that was designed to be dirty (*n* = 64), or (b) the same computer lab when it was clean (*n* = 74). Similar experimental designs have been used in previous research to increase the salience of infectious disease risk (e.g., Tybur et al., 2011; Reynolds et al., 2014). Given the nature of the manipulation, we randomized condition assignment between sessions, rather than participants. If participants asked about the dirtiness of the room, the research assistant explained that a taste-tasting study was conducted in the room earlier in the day and there was not enough time to clean up afterward. Participants were told that, because of time constraints, they were not allowed to clean up their individual computer stations.

Once in the experimental room, participants completed several computerized cognitive tasks, as well as the target dependent measure. Next, 30 min after entering the experimental room, participants provided an additional 5 mL saliva sample (Time 2), and then completed a series of measures collected for the purpose of a separate project. Finally, participants were thanked, debriefed, and awarded credit.

##### Dirty room condition

We prepared the testing room 30 min before each session. First, an unpleasant smell was produced by microwaving one cup of frozen broccoli florets for 5 min in a small amount of water about 10 min prior to each session. The keyboard and mouse at each computer were replaced with identical models that we covered in rubber cement, popcorn pieces, and a single strand of human hair. At each individual computer terminal, we also left food wrappers and water bottles (with a small amount of water left and food pieces floating therein). The two trash receptacles in the room were filled to the point of overflowing with discarded water bottles and food wrappers. Finally, the research assistant running each session wore a lab coat that was stained and wrinkled.

##### Clean room manipulation

In addition to making sure no visual or olfactory cues from the dirty room were present in the clean experimental room, we also took additional steps to increase perceptions of the room’s cleanliness. First, we removed all trash receptacles in the room. Next, we wiped down each keyboard and mouse with Lysol wipes (the original keyboards in the room were used, not the alternatives used in the dirty room condition), and placed a large bottle of hand sanitizer near the sign-in sheet. Finally, the research assistant running each session wore a clean, pressed lab coat.

##### Manipulation check

We used the following three items as a manipulation check of perceptions of room cleanliness: “The environment in this room: is pleasing to the eye; appears clean and sanitary; is organized and tidy.” All items were answered on the following scale: 1: *not at all*; 4: *somewhat*; 7: *completely*.

##### Levels of interleukin-1 beta

As a measure of participants’ acute immune activation, we collected their saliva just prior to (Time 1), and 30 min after (Time 2), entering the experimental room. Saliva was immediately frozen at −80°C after being collected. Saliva samples were later thawed, centrifuged, and assayed for levels of IL-1β, a proinflammatory cytokine, in duplicate using commercially available enzyme-linked immunosorbent assay (ELISA) kits (Salimetrics, Carlsbad, CA, United States) per manufacturer instructions. The intra-assay coefficient of variation (CV) was (2.45%); the inter-assay CV was (6.27%). We chose IL-1β as our analyte of interest both because of its role as the “quintessential proinflammatory cytokine” (Dinarello, 1984, 1991, 1997), as well as because it is one of the only proinflammatory cytokines for which well-validated, saliva-based ELISAs currently exist.

##### Probabilistic selection task

Participants completed the PST, a well-validated measure of reward and punishment sensitivity (Frank et al., 2004; Waltz et al., 2007; Whitmer et al., 2012), presented on Inquisit Experimental Software (version 4; Chantilly, VA). This task consists of both a learning phase and a test phase. For the learning phase, participants were presented with three stimulus pairs (hiragana symbols): AB, CD, EF. The position of each symbol on the right or left side of the screen was randomized between presentation; the hiragana symbol associated with each reward probability was randomized across participants. Participants were instructed to choose the “winning” symbol in each pair. Participants pressed the “A” key to select the symbol on the left, and the “L” key to select the symbol on the right. When the winning symbol was selected, the word “Correct!” was displayed in green font, accompanied by a sound of coins being deposited. If the losing symbol was selected, the word, “Wrong!” was displayed in red font, accompanied by a buzz. Feedback was displayed for 1,000 ms. Each symbol was associated with a different reward probability: A (80%) vs. B (20%), C (70%) vs. D (30%), and E (60%) vs. F (40%). The learning phase consisted of 30 trials (10 per symbol pair). Criteria for learning were selecting A at least 65% of the time, selecting C at least 60% of the time, and selecting E at least 50% of the time (with more difficult discrimination requiring less difficult criterion; Frank et al., 2004; Waltz et al., 2007; Whitmer et al., 2012). If participants did not meet these criteria, the learning phase was repeated until these criteria were met.

The tendency to choose A over B could be driven by the tendency to prefer A (due to positive feedback) or the tendency to avoid B (due to negative feedback). To parse these potential pathways, during the test phase, novel pairs of stimuli were presented in random order for AC, AD, AE, AF, BC, BD, BE, and BF across 160 trials (20 per symbol pair) with no feedback. We quantified reward sensitivity as a mean composite of the percent of times participants correctly chose A in the novel pairs (i.e., correctly chose the symbol rewarded at a higher probability). Punishment sensitivity was defined by the percent of times the participant correctly avoided choosing B in the novel pairs (i.e., correctly avoided the symbol punished at a higher probability).

##### Covariates

We measured a number of additional variables that may impact participants’ inflammatory responses, sensitivity to reward/punishment, or both. These included sex, age, body mass index (BMI), whether or not the participant regularly took anti-inflammatory medications (e.g., ibuprofen or aspirin), adult and childhood socioeconomic status (SES; Griskevicius et al., 2011), and the number of hours the participant slept the night before the session. We also administered the Positive and Negative Affect Schedule (Watson et al., 1988) to participants at the end of the study.

### Data Analysis Plan

First, we examined the data for normality and violations of common statistical assumptions. IL-1β levels were positively skewed, and the IL-1β data between groups (dirty vs. clean room) violated homogeneity of variances assumptions (Box’s test: *p* < 0.001; Levene’s test: *p* = 0.04 for Time 2 levels; see Table 2 for descriptive statistics). All other data were normally distributed. To account for the non-normal IL-1β data, we estimated our model with MLR estimation, which is robust to skewed variable distribution (Kline, 2016; Maydeu-Olivares, 2017), as well as Bayes estimation. We also examined whether demographic characteristics significantly differed between the two experimental groups. To test whether room condition predicted post-manipulation levels of salivary IL-1β controlling for Time 1 levels, we regressed Time 2 levels of IL-1β on both Time 1 levels, as well as room condition.

**TABLE 2:** Characteristics of the sample for Study 2.

	*M* (*SD*)	
Variable	Clean room (*n* = 74)	Dirty room (*n* = 64)
Sex	*M* = 21; *F* = 53	*M* = 42; *F* = 22
Age	19.04 (1.07)	18.95 (1.24)
BMI (kg/m^2^)	22.43 (2.93)	23.43 (3.32)
Adult SES (1–7)	5.18 (1.47)	5.07 (1.40)
Childhood SES (1–7)	5.34 (1.36)	5.53 (1.35)
Hrs Sleep	6.82 (1.59)	7.04 (1.52)

**BMI, body mass index; SES, socioeconomic status. Hours of sleep reported as the length of time participants slept the night before the session.**

The PST data did not violate any statistical assumptions. However, to remain consistent in our conservative statistical analysis and reporting, we again estimated the remaining models both with MLR estimation, as well as Bayesian estimation in MPlus. We tested two separate models to examine relationships between room condition, inflammation, and performance on the PST task. First, we used multivariate regression and simultaneously regressed both participants’ reward sensitivity (i.e., preference for A in novel pairings) as well as punishment sensitivity (i.e., avoidance of B in novel pairings) on room condition.

As a follow-up analysis, we tested whether any effect of room condition on PST performance was mediated through levels of salivary IL-1β at Time 2. We considered this analysis exploratory for several reasons. First, our primary objective was to test whether contexts that elicit increases in inflammation also lead to reductions in punishment sensitivity. However, at this point, we do not have a clear prediction about which cytokine(s) or other inflammatory protein(s) mediate this effect. Therefore, any relationship found between room condition and reward or punishment sensitivity may be mediated by a host of other candidate immunological proteins (i.e., other than IL-1β, e.g., interleukin-6, tumor necrosis factor-alpha; Simen et al., 2006; Treadway et al., 2017). Second, although previous research suggests that changes in levels of salivary cytokines reliably capture experimental immune activation (e.g., stress; Walsh et al., 2016; Newton et al., 2017; La Fratta et al., 2018), it is uncertain whether cytokines in saliva themselves directly influence central nervous system activity. In other words, changes in salivary cytokines serve as a valid proxy of inflammatory activity in response to an experimental manipulation, but may not themselves directly predict downstream psychological and behavioral processes. The latter are likely mediated through other pathways (e.g., cytokines in blood crossing the blood-brain barrier or stimulating vagal afferents; Konsman et al., 2002; Banks, 2005; Johnston and Webster, 2009). Lastly, to allow participants to interact with the room prior to measuring Time 2 IL-1β levels, post-manipulation saliva was collected just after, not before, participants completed the dependent measure. We examined the mediating role of levels of IL-1β by regressing Time 2 IL-1β levels on Time 1 levels and room condition, and then also regressing reward and punishment sensitivity on both room condition and Time 2 IL-1β levels.

We also tested each model a second time controlling for covariates (see section “Materials and Methods” for full list of covariates). For analyses comparing two discrete groups (i.e., dirty vs. clean room) without additional predictors, we report effect sizes as Cohen’s *d.* As with the previous study, we report effect sizes for analyses with multiple predictors as Cohen’s *f*^2^.

### Results and Discussion

#### Demographic Comparison and Manipulation Check

We conducted a multivariate analysis of variance (MANOVA) to examine whether groups differed on any of the continuous demographic variables. Results revealed that the groups did not significantly differ in age (*p* = 0.66), BMI (*p* = 0.07), adult SES (*p* = 0.68), childhood SES (*p* = 0.47), or hours of sleep (*p* = 0.41). We conducted a *X*^2^ test to examine whether the two groups differed by sex. Results revealed that sex did differ by condition, *X*^2^(1, *N* = 138) = 19.19, *p* < 0.001. Specifically, there were a greater proportion of men in the dirty room condition (men = 42; women = 22) than the clean room condition (men = 21; women = 53). Given that the two conditions differed in the number of men and women included in each group, we tested for interactions between condition and sex.

We conducted an independent samples *t*-test to compare participants’ ratings of room cleanliness between the two experimental room conditions (dirty vs. clean). Results revealed that participants in the dirty room found the room significantly less clean than participants in the clean room [*M*_*dirty*_ = 3.94, *SD*_*dirty*_ = 1.57; *M*_*clean*_ = 4.76, *SD*_*clean*_ = 1.10, *t*(138) = -3.64, *p* ≤ 0.001, *d* = 0.62]. Next, we conducted a multivariate analysis of variance (MANOVA) to test whether positive or negative affect differed by experimental condition (measured by PANAS scores). Results revealed that neither positive affect [*M*_*dirty*_ = 2.23, *SD*_*dirty*_ = 0.84; *M*_*clean*_ = 2.44, *SD*_*clean*_ = 0.81, *F*(1, 137) = 2.23, *p* = 0.14, *d* = 0.25], nor negative affect [*M*_*dirty*_ = 1.61, *SD*_*dirty*_ = 0.55; *M*_*clean*_ = 1.63, *SD*_*clean*_ = 0.56, *F*(1, 137) = 0.05, *p* = 0.83, *d* = 0.04] differed by room condition.

#### Impact of Room Condition on Levels of IL-1β

Both Time 1 IL-1β levels, β = 0.83, *SE* = 0.07, *t* = 11.84, *p* < 0.001, 95% CI = [0.77, 0.86], and room condition, β = −0.10, *SE* = 0.05, *t* = −2.03, *p* = 0.04, 95% CI = [−0.20, −0.03], *f*^2^ = 0.04, significantly predicted Time 2 IL-1β levels. Specifically, participants with higher levels of IL-1β at Time 1 also had higher levels at Time 2. Further, participants in the dirty room had significantly higher levels of IL-1β at Time 2 than participants in the clean room. The pattern and significance of these results did not change when we controlled for covariates (main effect of T1 IL-1β levels: *p* ≤ 0.001; main effect of room condition: *p* = 0.036, *f*^2^ = 0.04). There was also no significant interaction between room condition and sex in predicting T2 levels of IL-1β (*p* = 0.64).

#### Impact of Room Condition on Reward and Punishment Sensitivity

**FIGURE 2:**
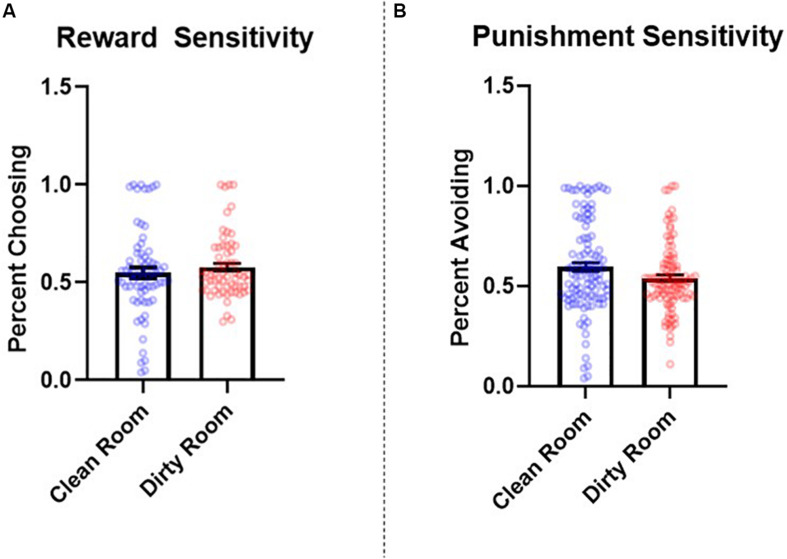
Between-subjects effects of room condition (clean room vs. dirty room) on **(A)** reward and **(B)** punishment sensitivity as measured by the probabilistic selection task in Study 2. Errors bars represent standard error of the mean.

The number of learning blocks required to reach each criterion did not differ significantly between experimental conditions (*p* = 0.62). Results revealed that there were no significant differences between groups in reward sensitivity, β = −0.08, *SE* = 0.08, *t* = −0.90, *p* = 0.37, 95% CI = [−0.24, 0.08], *d* = 0.14. However, punishment sensitivity was significantly lower in participants who completed the task in the dirty room compared to those in the clean room, β = 0.25, *SE* = 0.08, *t* = 3.17, *p* = 0.002, 95% CI = [0.05, 0.40], *d* = 0.52. See Figure 2 for a graph of these results. The pattern and significance of these results did not change when we controlled for covariates (reward sensitivity: *p* = 0.90, *f*^2^ = 0.001; punishment sensitivity: *p* = 0.007, *f*^2^ = 0.05). Further, we did not find evidence that IL-1β levels mediated relationships between room condition and either reward sensitivity (indirect effect: *p* = 0.92) or punishment sensitivity (indirect effect: *p* = 0.77). Sex did not interact with room condition to predict either reward sensitivity (*p* = 0.65) or punishment sensitivity (*p* = 0.26).

Study 2 found that participants who completed the study in a dirty room experienced a greater increase in salivary levels of IL-1β and were less sensitive to punishment (as measured by the PST) than those in a clean room. The current results suggest that contexts that elicit an increase in inflammatory activity may reduce the extent to which individuals alter their preferences in response to negative feedback. Although we did not find that levels of post-manipulation salivary IL-1β, specifically, mediated the relationship between room condition and performance on the PST, these results demonstrate that shifts in punishment sensitivity co-occur with heightened inflammatory activity.

## Study 3: Situational Suppression of Inflammation and Sensitivity to Reward and Punishment

Study 3 was designed to build on the results of Study 2 by further examining the role of immune activation – and inflammatory activity in particular – in regulating punishment sensitivity. While the results of Study 2 demonstrated that contexts that elicit an inflammatory response lead to a reduction in punishment sensitivity, we sought to test whether utilizing a manipulation designed to decrease inflammatory activity may have the opposite effect on this outcome. Specifically, we examined the effect of administering aspirin, an NSAID, (vs. a placebo) (Abramson et al., 1985; Weissmann, 1991; Yin et al., 1998; Morris et al., 2009; Elblbesy et al., 2012; Ortiz-Muñoz et al., 2014; Liu et al., 2017) – on reward and punishment sensitivity as measured by the PST. We predicted that participants who were administered aspirin would exhibit greater sensitivity to punishment than participants administered a placebo. However, consistent with what was observed in Study 2, we predicted that there would be no differences between the two groups in reward sensitivity.

### Materials and Methods

#### Participants

A total of 179 participants (109 women, *M*_*age*_ = 19.95 years, *SD*_*age*_ = 2.37, age range: 18–42 years) completed the study in exchange for partial course credit. Only participants who reported being free of chronic physical and psychological conditions were recruited for the study. All participants indicated that they could safely take the experimental and placebo medications at the time of signing up for the study and again at the time of consent. Participants were instructed to come into the lab fasting and abstain from taking recreational drugs (e.g., alcohol) and taking any prescription or over-the-counter anti-inflammatory medications for at least 48 h prior to participating. Due to a technical issue, eight participants were not able to complete the target dependent measure. Therefore, the final sample included 171 participants (109 women). In addition, 24 participants reported that they had taken either recreational drugs or administered an anti-inflammatory medication within 48 h prior to their session. We report results both with these latter participants included and excluded. see Table 4 for characteristics of the sample.

#### Materials and Procedure

This study was approved as ethical by Texas Christian University’s Institutional Review Board (DRB 1711-01). Participants arrived to the laboratory in the morning (between 8:00 and 9:30 a.m.). Participants provided informed consent and were seated at individually partitioned computer terminals. After providing consent, participants were randomly assigned to receive either only a placebo [*n* = 82; Vitamin B-6 (pyridoxine hydrochloride); 50 mg given as two 25 mg pills], or the same dose of a placebo (50 mg of Vitamin B-6; given as a single pill) plus a 325 mg dose of aspirin (*n* = 89). In both conditions, the medications were presented as two white, unmarked tablets.

After taking their assigned medications, participants filled out demographic questionnaires (including the same covariate measures used in Study 2) and watched a short, neutral filler video until 30 min had passed. This intermission was consistent with pharmacokinetic research into the length of time necessary for plasma levels of aspirin to peak following oral administration (Benedek et al., 1995). As in Study 2, participants then completed the same target PST task, followed by a number of additional cognitive tasks. At the conclusion of the session, participants were thanked, debriefed, and awarded credit.

### Data Analysis Plan

First, we examined the data for normality. Although the data did not violate normality or any statistical assumptions, to remain consistent, we again employed the conservative statistical approach used in the previous studies. Models were estimated in MPlus both with MLR and Bayesian estimation with non-informed priors (to produce CIs). We also examined whether demographic characteristics significantly differed between the two experimental groups. Participants’ reward sensitivity and punishment sensitivity were simultaneously regressed on condition (placebo vs. aspirin). The primary model was tested again controlling for potential covariates (see section “Materials and Methods” for full list of covariates). As in the previous studies, we report effect sizes as Cohen’s *d* for comparisons between discrete groups without additional predictors (i.e., aspirin group vs. placebo group) and report effect sizes as Cohen’s *f*^2^ for analyses with multiple predictors. We also estimated the primary model a second time with participants who reported not complying with study requirements (i.e., abstaining from recreational drugs or anti-inflammatory medications for the 48 h prior to their session) excluded.

### Results and Discussion

#### Demographic Comparison

We conducted a MANOVA to examine whether groups differed on any of the continuous demographic variables. Results revealed that groups did not significantly differ by age (*p* = 0.75), BMI (*p* = 0.21), adult SES (*p* = 0.70), childhood SES (*p* = 0.15), or hours of sleep (*p* = 0.15). We conducted a *X*^2^*-*test to examine whether the two groups differed by sex. Results revealed that that the groups did not significantly differ by sex (*p* = 0.15). Nonetheless, as in the first study, we examined sex differences in the impact of experimental condition on reward and punishment sensitivity.

#### Impact of Aspirin on Reward and Punishment Sensitivity

**FIGURE 3:**
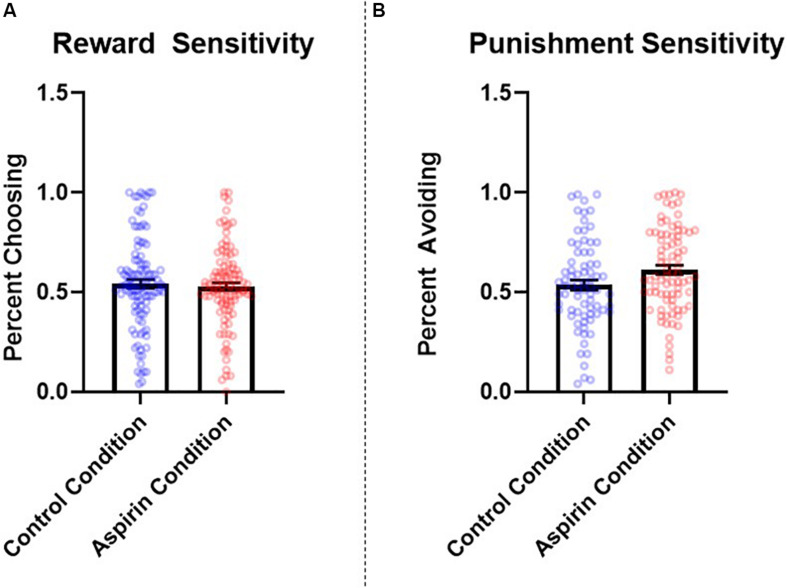
Between-subjects effects of condition (placebo vs. aspirin) on **(A)** reward and **(B)** punishment sensitivity as measured by the probabilistic selection task in Study 3. Errors bars represent standard error of the mean.

The number of learning blocks required to reach each criterion did not differ significantly between experimental conditions (*p* = 0.66). There were no significant differences between groups in reward sensitivity, β = −0.07, *SE* = 0.08, *t* = −0.90, *p* = 0.37, 95% CI = [−0.08, 0.05], *d* = 0.14. However, participants in the aspirin group exhibited significantly higher punishment sensitivity compared to participants in the placebo group, β = 0.18, *SE* = 0.08, *t* = 2.32, *p* = 0.02, 95% CI = [0.03, 0.33], *d* = 0.35. See Figure 3 for a graph of these results. The pattern and significance of the effect of condition on punishment sensitivity did not change when we excluded participants who did not comply with study requirements (see section “Participants” in “Materials and Methods;” reward sensitivity: *p* = 0.33, *d* = 0.15; punishment sensitivity: *p* = 0.025, *d* = 0.39), nor did they change when we controlled for covariates (see section “Materials and Methods” for full list; reward sensitivity*: p* = 0.57, *f*^2^ = 0.002; punishment sensitivity: *p* = 0.045, *f*^2^ = 0.03). Condition did not significantly interact with sex to predict either reward sensitivity (*p* = 0.45) or punishment sensitivity (*p* = 0.54).

The results of Study 3 provide additional evidence for a role of immunological involvement in regulating punishment sensitivity. Specifically, participants in the aspirin condition were more sensitive to negative feedback, choosing B less often than those administered a placebo. These results suggest that, just as contexts that elicit immune activation may reduce punishment sensitivity (i.e., completing the study in a dirty room; Study 2), experimentally administering a manipulation designed to suppress inflammation may enhance it.

## General Discussion

In the current research, we investigated the role that the activities of the immune system play in regulating punishment sensitivity. Based on insights from research in psychoneuroimmunology (Maier and Watkins, 1998; Banks, 2005; Dantzer and Kelley, 2007; Lasselin et al., 2017; Draper et al., 2018), and RSFT (Real and Caraco, 1986; Lima and Dill, 1990; Houston, 1991; McNamara and Houston, 1992), we predicted that punishment sensitivity would decrease in contexts where inflammation is elevated and increase when inflammatory activity is diminished. This pattern was hypothesized to occur because in the context of heightened inflammation (a) an individual’s probability of survival is lower, lowering the payoffs one can expect from investing in future-oriented behaviors (e.g., Gassen et al., 2019a, b; Gassen and Hill, 2019), and (b) the immunometabolic constraints that occur in this context decrease one’s ability to inhibit dominant responses (see e.g., Lacourt et al., 2018; Treadway et al., 2019).

Preliminary support for this hypothesis was found across three studies. Study 1 revealed that an environmental factor that promotes inflammatory activity (i.e., high infectious disease burden; e.g., Zhu et al., 1999; Gattone et al., 2001; Nazmi et al., 2010; Thompson et al., 2014; Ferrucci and Fabbri, 2018) was associated with the use of harsher punishments for criminal offenses. Although there may be numerous contributors that play a role in the association between these variables, it is consistent with the hypothesis that inflammation should predict reduced sensitivity to punishment, as harsher punishments are required to modify the behavior of individuals with lower punishment sensitivity (compared to those with higher punishment sensitivity; Jean-Richard-Dit-Bressel et al., 2018; Marchant et al., 2018). In addition to providing initial support for the hypothesis that the activities of the immune system will predict meaningful differences in punishment sensitivity, these results suggest that this relationship could have implications for criminal justice outcomes.

Studies 2 and 3 found continued support for the hypothesized relationship between inflammatory activity and punishment sensitivity. Study 2 revealed that exposure to an environment that elicited increased inflammatory activity (measured via salivary IL-1β) led to diminished punishment sensitivity. The results of Study 3 found further support for this hypothesis, demonstrating that administering a manipulation designed to experimentally reduce inflammatory activity (via aspirin administration) led to an increase in punishment sensitivity. No difference in reward sensitivity was observed across these two studies. Taken together, these results suggest that the activities of the immune system – and inflammation in particular – play a role in regulating punishment sensitivity. Further, these results provide preliminary evidence that the relationship between punitive measures and infectious disease burden found in Study 1 may be driven by elevated inflammation leading to (a) decreased punishment sensitivity and (b) harsher punishments to compensate for reduced sensitivity to punishment.

Together, the results of the current research add to a growing body of work demonstrating an important role for the immune system in regulating processes involved in learning (see e.g., Depino et al., 2004; Huang and Sheng, 2010; Sartori et al., 2012). Further, the current work contributes to the body of research examining inflammatory activity and processes related to punishment sensitivity (see e.g., Pugh et al., 1998; Patil et al., 2003; Sparkman et al., 2005; Kohman et al., 2007; Harrison et al., 2016). The latter is particularly important given the inconsistent results found across previous studies using different methods. For example, some studies have found no association between states known to be associated with increased inflammatory activity and punishment sensitivity (Kunisato et al., 2012; Berghorst et al., 2013). Others have found that heightened inflammatory activity *increases* punishment sensitivity (Harrison et al., 2016), with participants exhibiting more punishment sensitivity on a monetary task after an inflammatory response had been elicited via typhoid vaccination. One potential explanation for the inconsistencies between this previous work and the results of the current studies is that they reflect differences in the magnitude of the inflammatory response elicited by our manipulation (i.e., Study 2; dirty room) and theirs (i.e., typhoid vaccine). The typhoid vaccination used in Harrison et al. (2016) research resulted in an average 250% increase in plasma levels of interleukin-6, a proinflammatory cytokine. Our much subtler contextual manipulation of inflammation, on the other hand, only revealed an average 191% increase (with a rather large standard deviation) in IL-1β levels in saliva. As such, one possibility is that inflammation exerts a dose-dependent effect on punishment sensitivity, where small increases in inflammation may impair punishment sensitivity (as found in the current work), and larger increases in inflammation may enhance it (as found in Harrison et al., 2016). Moreover, differences in the timing of the punishment sensitivity task between our study and Harrison et al. (2016) may also help explain the disparate findings. Participants in the current research completed the PST shortly after entering the dirty room. In contrast, the behavioral task in Harrison et al. (2016) study was administered 2.5–3.5 h after vaccination. Thus, this could indicate that the effects of proinflammatory cytokines on reward and punishment sensitivity are time-dependent. Future research should examine these possibilities.

Much of the previous research studying the impact of inflammation on punishment sensitivity has been conducted using non-human animals. Consistent with the findings reported here, this animal research suggests that inflammatory challenges often reduce performance on tasks related to punishment sensitivity, such as avoiding aversive stimuli (e.g., foot shocks or predators) and contextual fear conditioning (Pugh et al., 1998; Patil et al., 2003; Sparkman et al., 2005; Kohman et al., 2007; Adelman et al., 2017). Moreover, during acute infection, which is associated with heightened inflammatory activity, house finches exhibit reduced behavioral avoidance of predators (Adelman et al., 2017).

Inherent in the current work are several limitations. For example, although Study 1 found that states with a greater infectious disease burden exhibited harsher punishments, it is possible that this association reflects processes other than reduced punishment sensitivity in the context of heightened inflammatory activity. For example, in addition to offenders, judges in high pathogen areas are also exposed to greater infectious disease risk than those in less pathogen dense areas. Thus, high infectious disease burden may influence psychological characteristics of judges (e.g., impulsivity) that render them more oriented toward harsher sentencing. Exploring these and other possibilities will be an important direction for future research.

The experimental studies also have important limitations. While Study 2 provided evidence that our manipulation of room cleanliness resulted in heightened inflammation and decreased punishment sensitivity, results did not provide evidence that levels of IL-1β mediated the relationship between room condition and punishment sensitivity. This could be due to a variety of factors. First, we only measured one proinflammatory cytokine, IL-1β. Given that a host of different proinflammatory proteins coordinates the inflammation response, it is possible that the relationship between room condition and punishment sensitivity is driven by a proinflammatory protein that we did not measure. Second, saliva samples were collected 30 min after exposure to room condition. It is possible that participants’ levels of inflammation were declining at this time and may not have been representative of their inflammatory levels during the task. As such, this detracts from our ability to make causal inferences about the role that inflammation plays in calibrating punishment sensitivity. However, it bears noting that past research examining the influence of experimental manipulations that elicit an inflammatory response on behavior often do not test or report whether inflammation serves as a mediator (e.g., Eisenberger et al., 2010; Inagaki et al., 2012; Harrison et al., 2016). It is important that future studies report these mediation analyses to provide evidence for or against claims of causal relationships between inflammation and behavioral outcomes.

Another potential limitation of Study 2 was our measurement of IL-1β in participants’ saliva samples, as opposed to peripheral blood samples (e.g., plasma or serum). Research into the strength of correlations between salivary and plasma/serum levels of cytokines across different contexts has yielded mixed results (e.g., Cruz-Almeida et al., 2017; La Fratta et al., 2018; Lee et al., 2018), and overall, there is a paucity of research on the topic. However, our primary objective for measuring levels of IL-1β was to provide a manipulation check on the prediction that exposure to the dirty room (compared to the clean room) would lead to a rise in inflammation. Recent research suggests that salivary measures of inflammation are well-suited for this purpose (e.g., Walsh et al., 2016; Newton et al., 2017; La Fratta et al., 2018; Gassen et al., 2019a). The results of Study 2 should also be interpreted with caution given that there were unequal numbers of men and women between the two conditions. While sex was controlled for in the analyses and did not significantly interact with experimental condition to predict any outcome, this is still an important limitation to consider.

One unexpected difference in punishment sensitivity emerged between the control conditions in Studies 2 and 3. Specifically, punishment sensitivity was higher in the clean room condition of Study 2 than in the control condition of Study 3. While we cannot say for certain what accounted for these differences, there was heterogeneity in the methods and sample characteristics between the two studies that may have contributed to them. First, the testing rooms used for the control conditions in each study were not equivalent. Specifically, to increase perceptions of cleanliness in the clean room condition of Study 2, a number of steps were taken to increase the room’s cleanliness, including removing trash receptacles, wiping down all of the computers and keyboards with disinfectant wipes, and placing a large bottle of hand sanitizer near the sign-in sheet. Given that these extra steps were not taken in the second experiment (for which room cleanliness was not part of the manipulation), the room used for the control condition in Study 2 was even cleaner than that used for the control condition in Study 3. Accordingly, it is possible that differences in punishment sensitivity between the two control conditions (with higher sensitivity found in Study 2) can be attributed to greater cleanliness in the control condition for Study 2 compared to Study 3.

Further, in Study 2, before entering the experimental room, participants provided their initial saliva sample in a separate room. They were then transferred to the experimental room before completing the remainder of the study. This differs from the methodology utilized in Study 3, where the entire study was completed in a single room. Although it is unclear how these procedural differences may influence punishment sensitivity, they are worthy of note in this context. A final explanation for the differences in punishment sensitivity that emerged between these conditions could lie in differences between demographic characteristics of the samples. As is displayed in Tables 2, 3, childhood and adult SES for the sample in the clean room condition for Study 2 were higher than for the control condition in Study 3 (*d* = 0.37–0.40). We are not aware of extant research finding SES-based differences in performance on the probabilistic selection task, specifically. However, more generally, research finds that those from a lower SES environment exhibit a higher risk for certain behavioral problems (e.g., impulsivity: Griskevicius et al., 2011), for which reduced punishment sensitivity has been identified as part of the underlying psychological architecture (e.g., Potts et al., 2006).

**TABLE 3:** Descriptive statistics for cytokine data in Study 2.

	*M* (*SD*)	
Variable	Dirty room	Clean room
Time 1 IL-1β	279.68 (452.71)	362.06 (560.34)
Time 2 IL-1β	601.93 (946.16)	492.46 (624.93)
Change in IL-1β (%)	191.11 (530.78)	115.85 (225.93)

**IL-1β, interleukin-1 beta. Time 1 levels of IL-1β measured in saliva collected just before entering the experimental room. Time 2 IL-1β levels assessed in saliva collected just after participants finished the behavioral tasks. IL-1β shown here untransformed as pg/mL.**

**TABLE 4:** Characteristics of the sample for Study 3.

	*M* (*SD*)	
Variable	Placebo condition (*n* = 82)	Aspirin condition (*n* = 89)
Sex	*M* = 25; *F* = 57	*M* = 37; *F* = 52
Age	20.12 (1.90)	19.93 (2.79)
BMI (kg/m^2^)	24.50 (5.29)	23.74 (3.70)
Adult SES (1–7)	4.62 (1.58)	4.52 (1.48)
Childhood SES (1–7)	4.75 (1.55)	5.00 (1.39)
Hrs Sleep	6.43 (1.33)	6.72 (1.42)

**BMI, body mass index; SES, socioeconomic status. Hours of sleep reported as the length of time participants slept the night before the session.**

An important limitation in the current work arises from the lack of measurement of inflammatory markers in Studies 1 and 3. That is, while participants in Study 3 who ingested aspirin – which reliably inhibits inflammatory activity (Yin et al., 1998; Morris et al., 2009; Chen and Stark, 2017; Liu et al., 2017) – exhibited increased punishment sensitivity compared to those who received a placebo, we did not directly measure inflammatory markers. Given that aspirin primarily operates by inhibiting inflammation and its physiological sequelae, aspirin was chosen as an experimental manipulation of inflammation. However, we did not directly measure inflammation in Study 3. As such, we cannot conclude that our results were driven specifically by changes in inflammation; these findings should be considered preliminary and interpreted with caution. Further, given our use of archival data in Study 1, we do not have data bearing on state-wide inflammation levels in the US. Although past research finds that inflammatory levels are higher for individuals with a higher pathogen burden (e.g., Nazmi et al., 2010; Thompson et al., 2014), to our knowledge, no research has examined whether infectious disease burden at the state level is associated with aggregate levels of inflammation. Future research, containing direct measures of inflammatory activity, is needed to better understand the potential dose- and time-dependent impacts of increased and suppressed inflammatory activity on punishment sensitivity.

There are a number of variables other than inflammation that likely influence punishment sensitivity that were not measured in the current research. Although we did not find that positive or negative affect differed by room condition (Study 2), changes in general pathogen concern or disgust sensitivity may play a role in regulating punishment or reward sensitivity in this context. Future research would also benefit from measuring additional physiological mediators, such testosterone or cortisol, that previous research has shown to influence individuals’ sensitivity to positive and negative experiences (e.g., van Honk et al., 2003, 2004; Berghorst et al., 2013).

Lastly although our theoretical framework predicts that decreased punishment sensitivity in the context of elevated inflammation is driven by a reduced willingness to expend effort to avoid potential threats (Mishra and Lalumière, 2010; Muehlenbein et al., 2010; Filosa et al., 2016; Lacourt et al., 2018; Wang et al., 2019), this proposed mediator was not measured in the current research. Future research would benefit from testing this explicitly. For example, in the PST, successful punishment avoidance requires an individual to both identify the stimulus more likely to be punished, and also inhibit the tendency to perform a motor movement (i.e., key pressing) previously paired with a reward (Potts et al., 2006; Inzlicht and Schmeichel, 2012; Westbrook and Braver, 2015; Wypych et al., 2019). Successfully responding to a probabilistic reward, on the other hand, may be less effortful in that it does not involve a similar degree of inhibition. This explanation, however, is only speculative and additional research is needed to further explore the role of effort in each punishment and reward sensitivity.

Despite its limitations, the results of the current research suggest that the immune system may play a role in regulating punishment sensitivity. Further, these results provide initial support for the possibility that relationships between the activities of the immune system and punishment sensitivity have implications for criminal justice outcomes. This research lays the groundwork for future studies to further examine how inflammation influences reward and punishment sensitivity, and as a result, the myriad behaviors related to these constructs.

## Data Availability Statement

The data analyzed for this study are publicly available on the Open Science Framework: doi: 10.17605/OSF.IO/29H7V.

## Ethics Statement

The studies involving human participants were reviewed and approved by Texas Christian University’s Institutional Review Board. The patients/participants provided their written informed consent to participate in this study.

## Author Contributions

HB and SM collected the data for Study 2. JG and SM collected the data for Study 3. JG performed the data analysis. SH oversaw the project execution. All authors designed the project and drafted this version of the manuscript.

## Conflict of Interest

The authors declare that the research was conducted in the absence of any commercial or financial relationships that could be construed as a potential conflict of interest.

## Footnotes

1 www.cdc.gov

2 https://www.fbi.gov/services/cjis/ucr

## References

[B1] Abramson S. Korchak H. Ludewig R. Edelson H. Haines K. Levin R. I. (1985). Modes of action of aspirin-like drugs. *Proc. Natl. Acad. Sci. U.S.A.* 82 7227–7231. 2997778 10.1073/pnas.82.21.7227 PMC390822

[B2] Adelman J. S. Mayer C. Hawley D. M. (2017). Infection reduces anti-predator behaviors in house finches. *J. Avian Biol.* 48 519–528. 10.1111/jav.01058 29242677 PMC5724792

[B3] Aubert A. (1999). Sickness and behaviour in animals: a motivational perspective. *Neurosci. Biobehav. Rev.* 23 1029–1036. 10.1016/s0149-7634(99)00034-2 10580315

[B4] Balleine B. W. (2011). “Sensation, incentive learning, and the motivational control of goal-directed action,” in *Neurobiology of Sensation and Reward*, ed. Gottfried J. A. (Boca Raton, FL: CRC Press), 287–311. 22593900

[B5] Banks W. A. (2005). Blood-brain barrier transport of cytokines: a mechanism for neuropathology. *Curr. Pharm. Design* 11 973–984. 10.2174/1381612053381684 15777248

[B6] Benedek I. H. Joshi A. S. Pieniaszek H. J. King S. Y. P. Kornhauser D. M. (1995). Variability in the pharmacokinetics and pharmacodynamics of low dose aspirin in healthy male volunteers. *J. Clin. Pharmacol.* 35 1181–1186. 10.1002/j.1552-4604.1995.tb04044.x 8750369

[B7] Benveniste E. N. (1992). Inflammatory cytokines within the central nervous system: sources, function, and mechanism of action. *Am. J. Physiol. Cell Physiol.* 263 C1–C16. 10.1152/ajpcell.1992.263.1.C1 1636671

[B8] Berghorst L. H. Bogdan R. Frank M. J. Pizzagalli D. A. (2013). Acute stress selectively reduces reward sensitivity. *Front. Human Neurosci.* 7:133. 10.3389/fnhum.2013.00133 23596406 PMC3622896

[B9] Bouton M. E. (2007). *Learning and Behavior: A Contemporary Synthesis.* Sunderland, MA: Sinauer Associates.

[B10] Boyle C. C. Kuhlman K. R. Dooley L. N. Haydon M. D. Robles T. F. Ang Y. S. (2019). Inflammation and dimensions of reward processing following exposure to the influenza vaccine. *Psychoneuroendocrinology* 102 16–23. 10.1016/j.psyneuen.2018.11.024 30496908 PMC6420390

[B11] Chen J. Stark L. A. (2017). Aspirin prevention of colorectal cancer: focus on NF-κB signalling and the nucleolus. *Biomedicines* 5:43. 10.3390/biomedicines5030043 28718829 PMC5618301

[B12] Cruz-Almeida Y. Aguirre M. Sorenson H. Tighe P. Wallet S. M. Riley J. L. III (2017). Age differences in salivary markers of inflammation in response to experimental pain: does venipuncture matter? *J. Pain Res.* 10 2365–2372. 10.2147/JPR.S138460 29042812 PMC5633270

[B13] Dantzer R. Kelley K. W. (2007). Twenty years of research on cytokine-induced sickness behavior. *Brain Behav. Immunity* 21 153–160. 10.1016/j.bbi.2006.09.006 17088043 PMC1850954

[B14] Depino A. M. Alonso M. Ferrari C. del Rey A. Anthony D. Besedovsky H. (2004). Learning modulation by endogenous hippocampal IL−1: blockade of endogenous IL−1 facilitates memory formation. *Hippocampus* 14 526–535. 10.1002/hipo.10164 15224987

[B15] Dinarello C. A. (1984). Interleukin-1. *Rev. Infect. Dis.* 6 51–95. 6369481 10.1093/clinids/6.1.51

[B16] Dinarello C. A. (1991). Interleukin-1 and interleukin-1 antagonism. *Blood* 77 1627–1652. 10.1182/blood.v77.8.1627.bloodjournal7781627 1826616

[B17] Dinarello C. A. (1997). Interleukin-1. *Cytokine Growth Factor Rev.* 8 253–265. 9620641 10.1016/s1359-6101(97)00023-3

[B18] Dong G. Huang J. Du X. (2011). Enhanced reward sensitivity and decreased loss sensitivity in Internet addicts: an fMRI study during a guessing task. *J. Psychiatric Res.* 45 1525–1529. 10.1016/j.jpsychires.2011.06.017 21764067

[B19] Dozmorov M. G. Bilbo S. D. Kollins S. H. Zucker N. Do E. K. Schechter J. C. (2018). Associations between maternal cytokine levels during gestation and measures of child cognitive abilities and executive functioning. *Brain Behav. Immunity* 70 390–397. 10.1016/j.bbi.2018.03.029 29588230 PMC6471612

[B20] Draper A. Koch R. M. van der Meer J. W. Apps M. A. Pickkers P. Husain M. (2018). Effort but not reward sensitivity is altered by acute sickness induced by experimental endotoxemia in humans. *Neuropsychopharmacology* 43 1107–1118. 10.1038/npp.2017.231 28948979 PMC5854801

[B21] Eisenberger N. I. Berkman E. T. Inagaki T. K. Rameson L. T. Mashal N. M. Irwin M. R. (2010). Inflammation-induced anhedonia: endotoxin reduces ventral striatum responses to reward. *Biol. Psychiatry* 68 748–754. 10.1016/j.biopsych.2010.06.010 20719303 PMC3025604

[B22] Elblbesy M. A. Hereba A. R. M. Shawki M. M. (2012). Effects of aspirin on rheological properties of erythrocytes in vitro. *Int. J. Biomed. Sci.* 8 188–193. 23675272 PMC3615281

[B23] Eppig C. Fincher C. L. Thornhill R. (2011). Parasite prevalence and the distribution of intelligence among the states of the USA. *Intelligence* 39 155–160. 10.1016/j.intell.2011.02.008 10955980

[B24] Erta M. Quintana A. Hidalgo J. (2012). Interleukin-6, a major cytokine in the central nervous system. *Int. J. Biol. Sci.* 8 1254–1266. 10.7150/ijbs.4679 23136554 PMC3491449

[B25] Farmer R. F. (2005). Temperament, reward and punishment sensitivity, and clinical disorders: implications for behavioral case formulation and therapy. *Int. J. Behav. Consult. Ther.* 1 56–76. 10.1037/h0100735

[B26] Ferrucci L. Fabbri E. (2018). Inflammageing: chronic inflammation in ageing, cardiovascular disease, and frailty. *Nat. Rev. Cardiol.* 15 505–522. 10.1038/s41569-018-0064-2 30065258 PMC6146930

[B27] Filosa A. Barker A. J. Dal Maschio M. Baier H. (2016). Feeding state modulates behavioral choice and processing of prey stimuli in the zebrafish tectum. *Neuron* 90 596–608. 10.1016/j.neuron.2016.03.014 27146269

[B28] Fincher C. L. Thornhill R. (2012). Parasite-stress promotes in-group assortative sociality: the cases of strong family ties and heightened religiosity. *Behav. Brain Sci.* 35 61–79. 10.1017/S0140525X11000021 22289223

[B29] Frank M. J. Seeberger L. C. O’reilly R. C. (2004). By carrot or by stick: cognitive reinforcement learning in parkinsonism. *Science* 306 1940–1943. 10.1126/science.1102941 15528409

[B30] Gassen J. Hill S. E. (2019). Why inflammation and the activities of the immune system matter for social and personality psychology (and not only for those who study health). *Soc. Pers. Psychol. Compass* 13:e12471.

[B31] Gassen J. Makhanova A. Maner J. K. Plant E. A. Eckel L. A. Nikonova L. (2019a). Experimentally-induced inflammation predicts present focus. *Adapt. Human Behav. Physiol.* 5 148–163. 10.1007/s40750-019-00110-7

[B32] Gassen J. Prokosch M. L. Eimerbrink M. J. Leyva R. P. P. White J. D. Peterman J. L. (2019b). Inflammation predicts decision-making characterized by impulsivity, present focus, and an inability to delay gratification. *Sci. Rep.* 9:4928. 10.1038/s41598-019-41437-1 30894653 PMC6426921

[B33] Gattone M. Iacoviello L. Colombo M. Di Castelnuovo A. Soffiantino F. Gramoni A. (2001). Chlamydia pneumoniae and cytomegalovirus seropositivity, inflammatory markers, and the risk of myocardial infarction at a young age. *Am. Heart J.* 142 633–640. 10.1067/mhj.2001.118118 11579353

[B34] Griskevicius V. Tybur J. M. Delton A. W. Robertson T. E. (2011). The influence of mortality and socioeconomic status on risk and delayed rewards: a life history theory approach. *J. Pers. Soc. Psychol.* 100 1015–1026. 10.1037/a0022403 21299312 PMC3298774

[B35] Harms M. B. Shannon Bowen K. E. Hanson J. L. Pollak S. D. (2018). Instrumental learning and cognitive flexibility processes are impaired in children exposed to early life stress. *Dev. Sci.* 21:e12596. 10.1111/desc.12596 29052307 PMC5908766

[B36] Harrington J. R. Gelfand M. J. (2014). Tightness–looseness across the 50 united states. *Proc. Natl. Acad. Sci. U.S.A.* 111 7990–7995. 10.1073/pnas.1317937111 24843116 PMC4050535

[B37] Harrison N. A. Brydon L. Walker C. Gray M. A. Steptoe A. Critchley H. D. (2009). Inflammation causes mood changes through alterations in subgenual cingulate activity and mesolimbic connectivity. *Biol. Psychiatry* 66 407–414. 10.1016/j.biopsych.2009.03.015 19423079 PMC2885494

[B38] Harrison N. A. Voon V. Cercignani M. Cooper E. A. Pessiglione M. Critchley H. D. (2016). A neurocomputational account of how inflammation enhances sensitivity to punishments versus rewards. *Biol. Psychiatry* 80 73–81. 10.1016/j.biopsych.2015.07.018 26359113 PMC4918729

[B39] Houston A. I. (1991). Risk−sensitive foraging theory and operant psychology. *J. Exp. Anal. Behav.* 56 585–589. 10.1901/jeab.1991.56-585 1774546 PMC1323140

[B40] Huang Z. B. Sheng G. Q. (2010). Interleukin-1β with learning and memory. *Neurosci. Bull.* 26 455–468. 10.1007/s12264-010-6023-5 21113196 PMC5560336

[B41] Inagaki T. K. Muscatell K. A. Irwin M. R. Cole S. W. Eisenberger N. I. (2012). Inflammation selectively enhances amygdala activity to socially threatening images. *Neuroimage* 59 3222–3226. 10.1016/j.neuroimage.2011.10.090 22079507 PMC3348143

[B42] Inzlicht M. Schmeichel B. J. (2012). What is ego depletion? Toward a mechanistic revision of the resource model of self-control. *Perspect. Psychol. Sci.* 7 450–463. 10.1177/1745691612454134 26168503

[B43] Iwasaki A. Medzhitov R. (2010). Regulation of adaptive immunity by the innate immune system. *Science* 327 291–295. 10.1126/science.1183021 20075244 PMC3645875

[B44] Janeway C. A. Jr. Medzhitov R. (2002). Innate immune recognition. *Annu. Rev. Immunol.* 20 197–216. 11861602 10.1146/annurev.immunol.20.083001.084359

[B45] Jappe L. M. Frank G. K. Shott M. E. Rollin M. D. Pryor T. Hagman J. O. (2011). Heightened sensitivity to reward and punishment in anorexia nervosa. *Int. J. Eating Disord.* 44 317–324. 10.1002/eat.20815 21472750 PMC3072848

[B46] Jean-Richard-Dit-Bressel P. Killcross S. McNally G. P. (2018). Behavioral and neurobiological mechanisms of punishment: implications for psychiatric disorders. *Neuropsychopharmacology* 43 1639–1650. 10.1038/s41386-018-0047-3 29703994 PMC6006171

[B47] Johnston G. R. Webster N. R. (2009). Cytokines and the immunomodulatory function of the vagus nerve. *Br. J. Anaesthesia* 102 453–462. 10.1093/bja/aep037 19258380

[B48] Kaeble D. Cowhig M. (2016). Correctional populations in the United States, 2016. *Bureau Justice Stat.* NCJ 251211.

[B49] Kavaliers M. Colwell D. D. (1995). Decreased predator avoidance in parasitized mice: neuromodulatory correlates. *Parasitology* 111 257–263. 10.1017/s0031182000081816 7567094

[B50] Kim S. H. Yoon H. Kim H. Hamann S. (2015). Individual differences in sensitivity to reward and punishment and neural activity during reward and avoidance learning. *Soc. Cogn. Affect. Neurosci.* 10 1219–1227. 10.1093/scan/nsv007 25680989 PMC4560942

[B51] Kipnis J. (2018). Immune system: the “seventh sense”. *J. Exp. Med.* 215 397–398. 10.1084/jem.20172295 29339443 PMC5789422

[B52] Kline R. B. (2016). *Principles and Practice of Structural Equation Modeling.* New York, NY: Guilford publications.

[B53] Kohman R. A. Tarr A. J. Byler S. L. Boehm G. W. (2007). Age increases vulnerability to bacterial endotoxin-induced behavioral decrements. *Physiol. Behav.* 91 561–565. 10.1016/j.physbeh.2007.03.032 17499821

[B54] Konsman J. P. Parnet P. Dantzer R. (2002). Cytokine-induced sickness behaviour: mechanisms and implications. *Trends Neurosci.* 25 154–159. 10.1016/s0166-2236(00)02088-9 11852148

[B55] Kunisato Y. Okamoto Y. Ueda K. Onoda K. Okada G. Yoshimura S. (2012). Effects of depression on reward-based decision making and variability of action in probabilistic learning. *J. Behav. Ther. Exp. Psychiatry* 43 1088–1094. 10.1016/j.jbtep.2012.05.007 22721601

[B56] La Fratta I. Tatangelo R. Campagna G. Rizzuto A. Franceschelli S. Ferrone A. (2018). The plasmatic and salivary levels of IL-1β, IL-18 and IL-6 are associated to emotional difference during stress in young male. *Sci. Rep.* 8:3031. 10.1038/s41598-018-21474-y PMC5813044 29445205

[B57] Lacourt T. E. Vichaya E. G. Chiu G. S. Dantzer R. Heijnen C. J. (2018). The high costs of low-grade inflammation: persistent fatigue as a consequence of reduced cellular-energy availability and non-adaptive energy expenditure. *Front. Behav. Neurosci.* 12:78. 10.3389/fnbeh.2018.00078 29755330 PMC5932180

[B58] Lasselin J. Treadway M. T. Lacourt T. E. Soop A. Olsson M. J. Karshikoff B. (2017). Lipopolysaccharide alters motivated behavior in a monetary reward task: a randomized trial. *Neuropsychopharmacology* 42 801–810. 10.1038/npp.2016.191 27620550 PMC5312062

[B59] Lee L. T. Wong Y. K. Hsiao H. Y. Wang Y. W. Chan M. Y. Chang K. W. (2018). Evaluation of saliva and plasma cytokine biomarkers in patients with oral squamous cell carcinoma. *Int. J. Oral Maxillofacial Surg.* 47 699–707. 10.1016/j.ijom.2017.09.016 29174861

[B60] Lima S. L. Dill L. M. (1990). Behavioral decisions made under the risk of predation: a review and prospectus. *Can. J. Zool.* 68 619–640. 10.1139/z90-092

[B61] Liu Y. Fang S. Li X. Feng J. Du J. Guo L. (2017). Aspirin inhibits LPS-induced macrophage activation via the NF-κB pathway. *Sci. Rep.* 7:11549. 10.1038/s41598-017-10720-4 PMC5599518 28912509

[B62] Lopes P. C. (2014). When is it socially acceptable to feel sick? *Proc. R. Soc. B Biol. Sci.* 281:20140218. 10.1098/rspb.2014.0218 24943375 PMC4083780

[B63] Maier S. F. Watkins L. R. (1998). Cytokines for psychologists: implications of bidirectional immune-to-brain communication for understanding behavior, mood, and cognition. *Psychol. Rev.* 105 83–107. 10.1037/0033-295x.105.1.83 9450372

[B64] Marchant N. J. Campbell E. J. Kaganovsky K. (2018). Punishment of alcohol-reinforced responding in alcohol preferring P rats reveals a bimodal population: implications for models of compulsive drug seeking. *Prog. Neuro Psychopharm. Biol. Psychiatry* 87 68–77. 10.1016/j.pnpbp.2017.07.020 28754407 PMC5785579

[B65] Matzinger P. (2002). The danger model: a renewed sense of self. *Science* 296 301–305. 10.1126/science.1071059 11951032

[B66] Maydeu-Olivares A. (2017). Maximum likelihood estimation of structural equation models for continuous data: standard errors and goodness of fit. *Struct. Equ. Model. Multidiscip. J.* 24 383–394. 10.1080/10705511.2016.1269606

[B67] McNamara J. M. Houston A. I. (1992). Risk-sensitive foraging: a review of the theory. *Bull. Math. Biol.* 54 355–378. 10.1016/s0092-8240(05)80031-x 18766164

[B68] Medzhitov R. Schneider D. S. Soares M. P. (2012). Disease tolerance as a defense strategy. *Science* 335 936–941. 10.1126/science.1214935 22363001 PMC3564547

[B69] Miller R. J. Jung H. Bhangoo S. K. White F. A. (2009). *“Cytokine and Chemokine Regulation of Sensory Neuron Function,” in Sensory Nerves.* Berlin: Springer, 417–449. 10.1007/978-3-540-79090-7_12 PMC2746245 19655114

[B70] Mishra S. Lalumière M. L. (2010). You can’t always get what you want: the motivational effect of need on risk-sensitive decision-making. *J. Exp. Soc. Psychol.* 46 605–611. 10.1016/j.jesp.2009.12.009

[B71] Mitchell J. T. Kimbrel N. A. Hundt N. E. Cobb A. R. Nelson−Gray R. O. Lootens C. M. (2007). An analysis of reinforcement sensitivity theory and the five−factor model. *Eur. J. Pers.* 21 869–887. 10.1002/per.644

[B72] Morris T. Stables M. Hobbs A. de Souza P. Colville-Nash P. Warner T. (2009). Effects of low-dose aspirin on acute inflammatory responses in humans. *J. Immunol.* 183 2089–2096. 10.4049/jimmunol.0900477 19597002

[B73] Muehlenbein M. P. Hirschtick J. L. Bonner J. Z. Swartz A. M. (2010). Toward quantifying the usage costs of human immunity: altered metabolic rates and hormone levels during acute immune activation in men. *Am. J. Human Biol.* 22 546–556. 10.1002/ajhb.21045 20309883

[B74] Muthén B. (2010). *Bayesian Analysis in Mplus: A Brief Introduction. Working Paper.* Available online at: http://www.statmodel.com/download/IntroBayesVersion%201.pdf (accessed May 2, 2010).

[B75] Muthén L. K. Muthén B. O. (2012). *MPlus User’s Guide*, 7th Edn Los Angeles, CA: Muthén & Muthén.

[B76] Nargeot R. Simmers J. (2011). Neural mechanisms of operant conditioning and learning-induced behavioral plasticity in Aplysia. *Cell. Mol. Life Sci.* 68 803–816. 10.1007/s00018-010-0570-9 21042832 PMC11114654

[B77] Nazmi A. Diez-Roux A. V. Jenny N. S. Tsai M. Y. Szklo M. Aiello A. E. (2010). The influence of persistent pathogens on circulating levels of inflammatory markers: a cross-sectional analysis from the Multi-Ethnic study of Atherosclerosis. *BMC Public Health* 10:706. 10.1186/1471-2458-10-706 21083905 PMC2996373

[B78] Newton T. L. Fernandez-Botran R. Lyle K. B. Szabo Y. Z. Miller J. J. Warnecke A. J. (2017). Salivary cytokine response in the aftermath of stress: an emotion regulation perspective. *Emotion* 17 1007–1020. 10.1037/emo0000156 28287751

[B79] O’Neill L. A. Kishton R. J. Rathmell J. (2016). A guide to immunometabolism for immunologists. *Nat. Rev. Immunol.* 16 553–565. 10.1038/nri.2016.70 27396447 PMC5001910

[B80] Opp M. R. (2005). Cytokines and sleep. *Sleep Med. Rev.* 9 355–364. 10.1016/j.smrv.2005.01.002 16102986

[B81] Ortiz-Muñoz G. Mallavia B. Bins A. Headley M. Krummel M. F. Looney M. R. (2014). Aspirin-triggered 15-epi-lipoxin A4 regulates neutrophil-platelet aggregation and attenuates acute lung injury in mice. *Blood* 124 2625–2634. 10.1182/blood-2014-03-562876 25143486 PMC4208278

[B82] Palminteri S. Justo D. Jauffret C. Pavlicek B. Dauta A. Delmaire C. (2012). Critical roles for anterior insula and dorsal striatum in punishment-based avoidance learning. *Neuron* 76 998–1009. 10.1016/j.neuron.2012.10.017 23217747

[B83] Patil C. S. Singh V. P. Satyanarayan P. S. V. Jain N. K. Singh A. Kulkarni S. K. (2003). Protective effect of flavonoids against aging-and lipopolysaccharide-induced cognitive impairment in mice. *Pharmacology* 69 59–67. 10.1159/000072357 12928578

[B84] Potts G. F. George M. R. M. Martin L. E. Barratt E. S. (2006). Reduced punishment sensitivity in neural systems of behavior monitoring in impulsive individuals. *Neurosci. Lett.* 397 130–134. 10.1016/j.neulet.2005.12.003 16378683

[B85] Preston D. L. Boland C. E. Hoverman J. T. Johnson P. T. (2014). Natural enemy ecology: comparing the effects of predation risk, infection risk and disease on host behaviour. *Funct. Ecol.* 28 1472–1481. 10.1111/1365-2435.12293

[B86] Pugh C. R. Kumagawa K. Fleshner M. Watkins L. R. Maier S. F. Rudy J. W. (1998). Selective effects of peripheral lipopolysaccharide administration on contextual and auditory-cue fear conditioning. *Brain Behav. Immunity* 12 212–229. 10.1006/brbi.1998.0524 9769157

[B87] Rae J. Murray D. (2019). Pathogen vs. predator: ranavirus exposure dampens tadpole responses to perceived predation risk. *Oecologia* 191 325–334. 10.1007/s00442-019-04501-1 31535255

[B88] Real L. Caraco T. (1986). Risk and foraging in stochastic environments. *Annu. Rev. Ecol. Syst.* 17 371–390. 10.1146/annurev.es.17.110186.002103

[B89] Reynolds L. M. McCambridge S. A. Bissett I. P. Consedine N. S. (2014). Trait and state disgust: an experimental investigation of disgust and avoidance in colorectal cancer decision scenarios. *Health Psychol.* 33 1495–1506. 10.1037/hea0000023 24447190

[B90] Santesso D. L. Dzyundzyak A. Segalowitz S. J. (2011). Age, sex and individual differences in punishment sensitivity: factors influencing the feedback−related negativity. *Psychophysiology* 48 1481–1489. 10.1111/j.1469-8986.2011.01229.x 21711354

[B91] Sartori A. C. Vance D. E. Slater L. Z. Crowe M. (2012). The impact of inflammation on cognitive function in older adults: implications for health care practice and research. *J. Neurosci. Nurs.* 44 206–217. 10.1097/jnn.0b013e3182527690 22743812 PMC3390758

[B92] Scott-Parker B. Watson B. King M. J. Hyde M. K. (2013). A further exploration of sensation seeking propensity, reward sensitivity, depression, anxiety, and the risky behaviour of young novice drivers in a structural equation model. *Accident Anal. Prevention* 50 465–471. 10.1016/j.aap.2012.05.027 22770376

[B93] Selya A. S. Rose J. S. Dierker L. C. Hedeker D. Mermelstein R. J. (2012). A practical guide to calculating Cohen’s f^2^, a measure of local effect size, from PROC MIXED. *Front. Psychol.* 3:111. 10.3389/fpsyg.2012.00111 22529829 PMC3328081

[B94] Shanks D. R. (1993). Human instrumental learning: a critical review of data and theory. *Br. J. Psychol.* 84 319–354. 10.1111/j.2044-8295.1993.tb02486.x 8401987

[B95] Simen B. B. Duman C. H. Simen A. A. Duman R. S. (2006). TNFα signaling in depression and anxiety: behavioral consequences of individual receptor targeting. *Biol. Psychiatry* 59 775–785. 10.1016/j.biopsych.2005.10.013 16458261

[B96] Sistad R. E. Simons R. M. Simons J. S. (2019). Sensitivity to reward and punishment and alcohol outcomes: metacognition as a moderator. *Addict. Behav. Rep.* 10:100213. 10.1016/j.abrep.2019.100213 31517019 PMC6728263

[B97] Skinner B. F. (1963). Operant behavior. *Am. Psychol.* 18:503.

[B98] Smith R. J. Laiks L. S. (2018). Behavioral and neural mechanisms underlying habitual and compulsive drug seeking. *Prog. Neuro Psychopharm. Biol. Psychiatry* 87 11–21. 10.1016/j.pnpbp.2017.09.003 28887182 PMC5837910

[B99] Sparkman N. L. Kohman R. A. Garcia A. K. Boehm G. W. (2005). Peripheral lipopolysaccharide administration impairs two-way active avoidance conditioning in C57BL/6J mice. *Physiol. Behav.* 85 278–288. 10.1016/j.physbeh.2005.04.015 15936787

[B100] Staddon J. E. Cerutti D. T. (2003). Operant conditioning. *Annu. Rev. Psychol.* 54 115–144. 12415075 10.1146/annurev.psych.54.101601.145124 PMC1473025

[B101] The Pew Charitable Trusts (2016). *Punishment Rate Measures Prison Use Relative to Crime.* Available online at: https://www.pewtrusts.org/en/research-and-analysis/issue-briefs/2016/03/the-punishment-rate (accessed March 23, 2016).

[B102] Thompson A. L. Houck K. M. Adair L. Gordon−Larsen P. Du S. Zhang B. (2014). Pathogenic and obesogenic factors associated with inflammation in Chinese children, adolescents and adults. *Am. J. Human Biol.* 26 18–28. 10.1002/ajhb.22462 24123588 PMC3932143

[B103] Thorndike E. L. (1898). *Animal Intelligence: Experimental Studies.* New York, NY: The Macmillan Company.

[B104] Thornhill R. Fincher C. L. (2011). Parasite stress promotes homicide and child maltreatment. *Philos. Trans. R. Soc. B Biol. Sci.* 366 3466–3477. 10.1098/rstb.2011.0052 22042922 PMC3189353

[B105] Treadway M. T. Admon R. Arulpragasam A. R. Mehta M. Douglas S. Vitaliano G. (2017). Association between interleukin-6 and striatal prediction-error signals following acute stress in healthy female participants. *Biol. Psychiatry* 82 570–577. 10.1016/j.biopsych.2017.02.1183 28506437 PMC5610086

[B106] Treadway M. T. Cooper J. A. Miller A. H. (2019). Can’t or won’t? Immunometabolic constraints on dopaminergic drive. *Trends Cogn. Sci.* 23 435–448. 10.1016/j.tics.2019.03.003 30948204 PMC6839942

[B107] Tybur J. M. Bryan A. D. Magnan R. E. Hooper A. E. C. (2011). Smells like safe sex: olfactory pathogen primes increase intentions to use condoms. *Psychol. Sci.* 22 478–480. 10.1177/0956797611400096 21350181

[B108] U.S. Census Bureau (2018). *American Community Survey 2013-2017 5-year Data Release.* Available online at: https://www.census.gov/newsroom/press-kits/2018/acs-5year.html (accessed December 6, 2018).

[B109] van Honk J. Schutter D. J. Hermans E. J. Putman P. (2003). Low cortisol levels and the balance between punishment sensitivity and reward dependency. *Neuroreport* 14 1993–1996. 10.1097/00001756-200310270-00023 14561936

[B110] van Honk J. Schutter D. J. Hermans E. J. Putman P. Tuiten A. Koppeschaar H. (2004). Testosterone shifts the balance between sensitivity for punishment and reward in healthy young women. *Psychoneuroendocrinology* 29 937–943. 10.1016/j.psyneuen.2003.08.007 15177710

[B111] Vichaya E. G. Hunt S. C. Dantzer R. (2014). Lipopolysaccharide reduces incentive motivation while boosting preference for high reward in mice. *Neuropsychopharmacology* 39 2884–2890. 10.1038/npp.2014.141 24917202 PMC4200499

[B112] Walsh E. Eisenlohr-Moul T. Baer R. (2016). Brief mindfulness training reduces salivary IL-6 and TNF-α in young women with depressive symptomatology. *J. Consult. Clin. Psychol.* 84 887–897. 10.1037/ccp0000122 27281371 PMC5037002

[B113] Waltz J. A. Frank M. J. Robinson B. M. Gold J. M. (2007). Selective reinforcement learning deficits in schizophrenia support predictions from computational models of striatal-cortical dysfunction. *Biol. Psychiatry* 62 756–764. 10.1016/j.biopsych.2006.09.042 17300757 PMC2083701

[B114] Wang A. Luan H. H. Medzhitov R. (2019). An evolutionary perspective on immunometabolism. *Science* 363:eaar3932. 10.1126/science.aar3932 30630899 PMC6892590

[B115] Watson D. Clark L. A. Tellegen A. (1988). Development and validation of brief measures of positive and negative affect: the PANAS scales. *J. Pers. Soc. Psychol.* 54 1063–1070. 10.1037/0022-3514.54.6.1063 3397865

[B116] Wegner A. Elsenbruch S. Maluck J. Grigoleit J. S. Engler H. Jäger M. (2014). Inflammation-induced hyperalgesia: effects of timing, dosage, and negative affect on somatic pain sensitivity in human experimental endotoxemia. *Brain Behav. Immunity* 41 46–54. 10.1016/j.bbi.2014.05.001 24814500

[B117] Weissmann G. (1991). Aspirin. *Sci. Am.* 264 84–91. 10.1038/scientificamerican0191-84 1899486

[B118] Westbrook A. Braver T. S. (2015). Cognitive effort: a neuroeconomic approach. *Cogn. Affect. Behav. Neurosci.* 15 395–415. 10.3758/s13415-015-0334-y 25673005 PMC4445645

[B119] Whitmer A. J. Frank M. J. Gotlib I. H. (2012). Sensitivity to reward and punishment in major depressive disorder: effects of rumination and of single versus multiple experiences. *Cogn. Emot.* 26 1475–1485. 10.1080/02699931.2012.682973 22716241 PMC11880990

[B120] Wypych M. Michałowski J. M. Droździel D. Borczykowska M. Szczepanik M. Marchewka A. (2019). Attenuated brain activity during error processing and punishment anticipation in procrastination–a monetary Go/no-go fMRi study. *Sci. Rep.* 9:11492. 10.1038/s41598-019-48008-4 31391541 PMC6685938

[B121] Yin M. J. Yamamoto Y. Gaynor R. B. (1998). The anti-inflammatory agents aspirin and salicylate inhibit the activity of IκB kinase-β. *Nature* 396 77–80. 10.1038/23948 9817203

[B122] Zhu J. Quyyumi A. A. Norman J. E. Csako G. Epstein S. E. (1999). Cytomegalovirus in the pathogenesis of atherosclerosis: the role of inflammation as reflected by elevated C-reactive protein levels. *J. Am. Coll. Cardiol.* 34 1738–1743. 10577564 10.1016/s0735-1097(99)00410-6

